# Marked Elevation of Lipase in COVID-19 Disease: A Cohort Study

**DOI:** 10.14309/ctg.0000000000000215

**Published:** 2020-07-16

**Authors:** Usman Barlass, Brett Wiliams, Klodian Dhana, Darbaz Adnan, Shahab R. Khan, Mahboobeh Mahdavinia, Faraz Bishehsari

**Affiliations:** 1 Division of Gastroenterology, Department of Internal Medicine, Rush University Medical Center, Chicago, Illinois, USA;; 2 Division of Infection Disease, Department of Internal Medicine, Rush University Medical Center, Chicago, Illinois, USA;; 3 Department of Internal Medicine, Rush University Medical Center, Chicago, Illinois, USA;; 4 Rush Institute for Healthy Aging, Rush University Medical Center, Chicago, Illinois, USA;; 5 Division of Allergy and Immunology, Department of Internal Medicine, Rush University Medical Center, Chicago, Illinois, USA.

## Abstract

**INTRODUCTION:**

Severe acute respiratory syndrome coronavirus 2 (SARS-CoV-2) causing the pandemic of coronavirus disease 2019 (COVID-19) is a global health crisis. Possible pancreatic involvement has recently been observed in these patients; however, its significance is unclear. The aim of this study was to evaluate the association of significantly elevated lipase with disease outcomes.

**METHODS:**

Data about demographics, symptoms, laboratory values, and clinical outcomes were collected for 1,003 consecutive patients testing positive for COVID-19. Elevated lipase was defined as greater than 3 times the upper limit of normal (>3 × ULN). Baseline characteristics among patients with or without elevated lipase were compared using Fisher exact test or Student *t*-test for categorical or numerical variables, respectively. Logistic regression was used to evaluate the association of lipase levels with primary clinical outcomes (intensive care unit admission and intubation) adjusted for age, sex, body mass index, history of diabetes, and hypertension.

**RESULTS:**

Of 1,003 patients with COVID-19, 83 had available lipase levels and were all admitted to the hospital. Of 83, 14 (16.8%) had elevated lipase (>3 × ULN), which was associated with higher rates of leukocytosis (*P* < 0.001) and abnormal liver enzymes (*P* < 0.01). Compared with lower lipase levels (<3 × ULN), patients with elevated lipase had higher rates of ICU admission (92.9% vs 32.8%; *P* < 0.001) and intubation (78.6% vs 23.5%; *P* 0.002). In a multivariable-adjusted model, higher lipase levels were significantly associated with admission to the ICU and rate of intubation.

**DISCUSSION:**

Lipase elevation is seen in COVID-19 and is associated with worse disease outcomes.

## INTRODUCTION

Severe acute respiratory syndrome coronavirus 2 (SARS-CoV-2) has caused the ongoing pandemic of coronavirus disease 2019 (COVID-19) leading to a global health crisis (1).

The entry of SARS-CoV-2 into cells is mediated via angiotensin-converting enzyme 2 (ACE2) receptor (2,3). Besides the respiratory epithelium which is a typical site for the virus entry, several other tissues including the gastrointestinal tract, liver, and pancreas express high levels of ACE2 (4). Therefore, COVID-19 may affect and cause inflammation in multiple organs (5), causing a plethora of clinical symptoms and laboratory abnormalities that are coupled with a more severe disease process (1). Worse outcomes from COVID-19 are often characterized by the need for intensive care unit (ICU) admission, fever from persistent systemic inflammation with or without superimposed infection, and multiple organ failure (6).

Elevated lipase may suggest pancreatic injury and has recently been described in up to 18% of patients admitted with severe COVID-19 in China (7). However, there are limited data on the significance of elevated lipase in COVID-19 (7,8). Pancreatic involvement in COVID-19 infection is of particular interest because the ACE2 receptor levels in the pancreas have been shown to be higher than in the respiratory epithelium (7). It is therefore possible that pancreatic injury may perpetuate systemic inflammation from COVID-19, associated with worse outcomes. Consistent with this hypothesis, and after our observation of elevated lipase in 3 cases with severe COVID-19 disease, we aimed to determine the significance of lipase levels on the clinical outcome of COVID-19 in a cohort of patients who consecutively tested positive for SARS-CoV-2 infection.

## METHODS

### Study population

Summary of our initial observation of 3 cases with severe COVID-19 disease is presented. For the cohort study, the inclusion criteria were all patients (age ≥18 years) who tested positive for SARS-CoV-2 by polymerase chain reaction nasopharyngeal swab at Rush University Medical Center between March 12 and April 3, 2020 (n = 1,003). Detailed laboratory data were available in those who were hospitalized (n = 294). Pancreatic lipase levels (normal range 10–52 U/L) were tested in 83 (18%) of these patients. High lipase was defined as being greater than 3 times the upper limit of normal (>3 × ULN; >156 U/L). Abnormal liver enzymes were defined as serum values greater than ULN, i.e., aspartate aminotransferase (AST) > 44 U/L, alanine aminotransferase (ALT) >40 U/L, and bilirubin >1.3 mg/dL. Patients were intubated and/or admitted to the ICU as clinically indicated at the discretion of the clinicians directly responsible for patients care.

Electronic encounters of all the patients were reviewed by trained researchers to record baseline demographics (age, sex, body mass index [BMI], history of diabetes, and hypertension), relevant clinical symptoms (fever, nausea, vomiting, abdominal pain, and diarrhea), laboratory data (lipase levels, white blood cell count, and liver enzymes: AST, ALT, bilirubin), and clinical end points (whether ICU admission was required and whether the patients required intubation for ventilatory support).

This study was approved and conducted under the institutional review board (IRB # 20040208-IRB01).

### Statistical analysis

Baseline characteristics of the study population are shown as the number (%) of participants or mean and SEM. Statistical differences of baseline characteristics among patients with or without elevated lipase levels (as defined above) were analyzed with the Fisher exact test or Student *t*-test for categorical or numerical variables, respectively. Logistic regression analyses were used to quantify the associations between lipase levels with COVID-19 complications, including intubation and/or admission to the ICU. Lipase levels were further analyzed as continuous and categorical variables. The continuous variable was log-transformed to stabilize the variance and to obtain normal distributions variables. The categorical variables were composed of 3 equal groups, tertiles, and the lowest tertile was used as a reference. The regression models were adjusted by age (years), sex (male vs female), BMI (kg/m^2^), history of diabetes (yes vs no), and history of hypertension (yes vs no). *P* value of < 0.05 was considered significant. All the analyses were performed using R software (CRAN version 3.6.0).

## RESULTS

### Three patients with severe COVID-19 disease and recurrent fever were found to have elevated lipase

#### Case 1

A middle-aged obese (BMI = 39) man presented with 14 days of flu-like symptoms and tested positive for COVID-19. He was intubated due to hypoxia on day 17 of illness. He became febrile on day 28 and was started on empiric broad-spectrum antibiotics, which were narrowed to cefazolin based on susceptibilities from sputum culture. Given persistent fever and leukocytosis despite targeted antibiotics, lipase was checked on days 31 and 33 and was 262 U/L and 587 U/L, respectively. Ultrasound demonstrated gallbladder sludge but no duct dilation. Abdominal tenderness could not be assessed given deep sedation. Polymerase chain reaction resulted negative for COVID-19 on day 35. However, the patient remained intubated with leukocytosis and intermittent fevers at day 43, despite several courses of varying broad-spectrum antibiotics and negative cultures.

#### Case 2

A young obese (BMI = 40) adult woman presented with 14 days of flu-like symptoms was tested positive for COVID-19 and was intubated in the emergency department for hypoxia. Hypoxia and high fever progressed despite empiric antibiotics and a dose of tocilizumab on day 24, which was repeated on day 29. Her fever curve improved, and she was weaned off of vasopressors. However, fever and leukocytosis recurred 2 days later, whereas infection workup remained negative. Lipase was checked on days 33 and 35 which were 300 U/L and 347 U/L, respectively. On day 42, CT angiogram which ruled out pulmonary embolus showed no biliary duct dilation; however, the pancreas was not imaged. Fever stopped after day 35 and leukocytosis resolved by day 43.

#### Case 3

A young woman with obesity (BMI = 49) presented to an outside hospital with shortness of breath and was intubated in their emergency room, and was diagnosed with COVID-19. After 3 days, she was transferred to our hospital and given a dose of tocilizumab for progressive hypoxia. Fever and leukocytosis began on day 11 of admission, and she was started on antibiotics for a presumed ventilator-associated pneumonia; leukocytosis resolved, but the need for respiratory support and recurrent fever persisted despite the lack of any infectious etiology and negative extremity Doppler ultrasound for thrombosis. Because of persistent fever, the lipase level was ordered on day 20 of admission and was 477 U/L. She remained intubated with spikes of fever 21 days after admission.

### Lipase elevation was associated with severe COVID-19 disease in our cohort study

Of 1,003 COVID-19-positive patients, lipase levels were available in patients who were admitted to the hospital. Among these 294 admitted patients, 83 (18%) were tested for lipase. Fourteen (of 83; 16.8%) had elevated lipase (see methods, and Table 1). There was a significant predominance of men in the elevated lipase group compared with the lower lipase group (78.6% vs 38.8%; *P* = 0.009). However, no other significant difference was observed in the demographics of the 2 groups (Table 1). Except higher symptoms of nausea or vomiting in the low lipase group (75.4% vs 50%; *P* < 0.025), there was no other significant difference in recorded gastrointestinal symptoms such as abdominal pain or diarrhea between the 2 groups (Table 1).

**Table 1.**
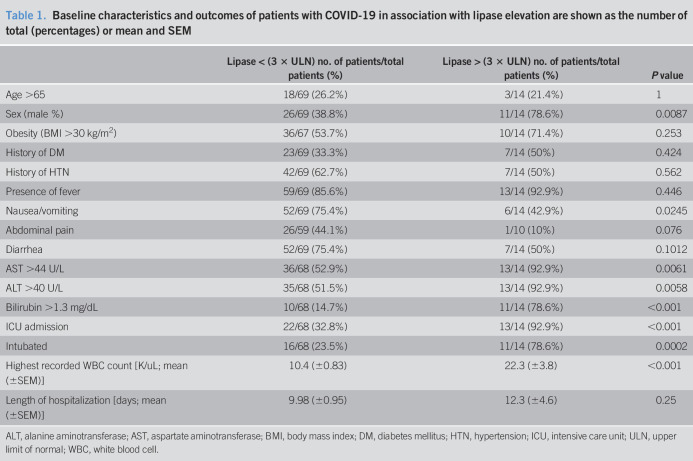
Baseline characteristics and outcomes of patients with COVID-19 in association with lipase elevation are shown as the number of total (percentages) or mean and SEM

Maximum recorded white count during the course of admission was significantly higher in the elevated lipase group (22.3 ± 3.8 vs 10.4 ± 0.83; *P* < 0.001). Likewise, patients with elevated lipase had higher rates of abnormal liver enzymes (AST, ALT, and bilirubin, *P* < 0.05) compared with the low lipase groups (Table 1).

Comparing the outcomes in the 2 groups revealed significantly higher rates of ICU admission (92.9% vs 32.8%; *P* < 0.001) and intubation rates (78.6% vs 23.5%; *P* = 0.0002) in patients with elevated (>3 ULN) lipase (Table 1). There was a trend for higher length of hospitalization in the elevated lipase group (mean 12.3 days vs 9.98 days); however, it did not reach statistical significance.

**Figure 1.**
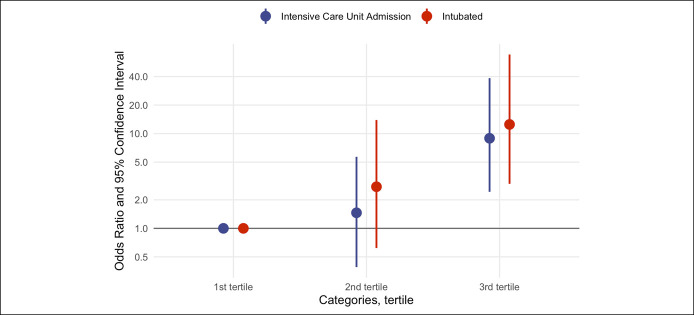
Odds ratio (with 95% confidence intervals) of rates of ICU admission and intubation based on the lipase levels divided in tertiles (first tertile–lipase level; 5–31 U/L, second tertile; 31–75 U/L, third tertile; 81–701 U/L). ICU, intensive care unit.

In the multivariable-adjusted model, elevated lipase level was significantly associated with the rates of admission to the ICU and intubation after adjusting for age, sex, BMI, history of diabetes, and history of hypertension (Table 2). For a one-unit increase in natural log-transformed lipase, the odds ratio of admission to the ICU was 2.75 (95% confidence interval [CI] 1.57–5.34; *P* = 0.001). Comparing with patients in the first tertile (lipase levels 5–31 U/L) those in the second tertile (lipase levels 32–75 U/L) and third tertile (lipase levels 81–701 U/L) had an odds ratio (95% CI) for ICU admission of 1.46 (0.39–5.69; *P* = 0.578) and 8.93 (2.43–38.5; *P* = 0.002), respectively (Table 2 and Figure 1).

**Table 2.**
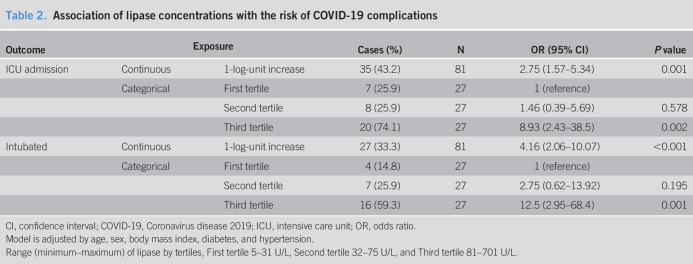
Association of lipase concentrations with the risk of COVID-19 complications

Similarly, for one-unit increase in natural log-transformed lipase, the odds ratio of intubation was 4.16 (95% CI 2.06–10.07). Compared with patients in the first tertile (lipase levels 5–31 U/L) those in the second and third tertile had an odds ratio (95% CI) for intubation of 2.75 (0.62–13.92; *P* = 0.195) and 12.5 (2.95–68.4; *P* = 0.001), respectively (Table 2 and Figure 1).

## DISCUSSION

ACE2 receptors are required for SARS-CoV-2 entry into cells (9), and recent studies have shown that the pancreatic tissue expresses this receptor (4,7). Although gastrointestinal manifestations of COVID-19 disease including nausea, vomiting, and abdominal pain have been described with a pooled prevalence of 17.6% in a meta-analysis of 60 studies and over 4,000 patients (10), little is known about the possible pancreatic involvement in the disease process.

Recent case reports from different parts of the world have presented acute pancreatitis as possible complication of SARS-CoV-2 infection (11–13). In addition, a recent study from China with 52 patients suggest possible pancreatic injury leading to lipase elevation in COVID-19 infection (7,8). Although the level of lipase elevation in this study was not specifically defined, the clinical significance of lipase elevation on the course of COVID-19 has not been addressed (14).

To the best of our knowledge, we present the largest study to date evaluating the association of lipase elevation with COVID-19 disease outcomes. We first observed the remarkable elevation of lipase in 3 cases of severe COVID-19 who needed ICU support and had persistent fever despite negative infectious workup. This led us to conduct our large retrospective cohort study where we found 16.8% of patients with COVID-19 who were tested for lipase had >3 ULN levels. Lipase testing was at the discretion of the clinical team. However, our results suggest that gastrointestinal symptoms were not the factor that prompted clinicians to check the lipase in these patients with COVID-19. Interestingly, elevated lipase was associated with worse outcomes defined by ICU admission and intubation rates. The association between lipase levels and these disease outcomes remained significant even after adjusting for multiple other confounders.

Pancreatic injury and inflammation are a well-known cause of organ failure with relapsing fever (15). Although pancreas involvement is reported in other viruses (16), it is unclear whether SARS-CoV-2 can directly affect the pancreas or the pancreas is involved as part of the virus-triggered systemic inflammatory response and cytokine storm leading to multiorgan dysfunction (14,17). Furthermore, there is an abundance of data regarding nonpancreatic causes of lipase elevation in critically ill patients from etiologies such as pancreatic hypoperfusion from shock, mechanical ventilation, pulmonary disease, and renal failure (18), any of which can occur in COVID-19 (1). Therefore, lipase elevation in COVID-19 may be due to pancreatic involvement or multiorgan dysfunction or both. Nevertheless, our data show that elevated lipase could be an independent marker of severe disease in COVID-19.

Further studies are needed to better assess the pancreas as the possible source of elevated lipase in COVID-19 and whether pancreatic injury could represent a distinct disease phenotype characterized by a worsened inflammatory profile and hence disease outcomes.

Limitations to our study include the lack of proper abdominal imaging of the pancreas to appropriately assess for pancreatic injury as a source of elevated lipase. As a result, we cannot exclude nonpancreatic sources of lipase elevation. Although the cutoff of 3 × ULN decreases that probability, lipase could be elevated from the leakage of digestive enzymes from the intestine as well (19). Intestinal tract alone, however, does not seem to explain the elevated lipase in our cohort, given the comparable frequency of symptoms (i.e., diarrhea) between the high and low lipase groups.

In conclusion, our study suggests an association of elevated lipase in COVID-19 disease with worse outcomes.

## CONFLICTS OF INTEREST

**Guarantor of the article:** Faraz Bishehsari, MD, PhD.

**Specific author contributions:** Study concept and design (B.W. and F.B.), acquisition of data (D.A., S.R.K., B.W., M.M., and F.B.), analysis and interpretation of data (U.B., K.D., and F.B.); drafting of the manuscript (B.W., U.B., and F.B.), and critical revision of the manuscript for important intellectual content (M.M. and F.B.).

**Financial support:** National Institutes of Health grant AA025387, Brinson foundation and a Rush Translational Sciences Consortium/Swim Across America Organization grant (F.B.), and KL2TR002387-02 (M.M.).

**Potential competing interests:** None to report.

**WHAT IS KNOWN**

✓ Receptor for SARS-CoV-2 is expressed in the pancreas.
✓ Pancreatic injury is reported in COVID-19 disease.
✓ The significance of lipase elevation in COVID-19 disease in unclear.

**WHAT IS NEW HERE**

✓ Elevated lipase is seen in severe COVID-19 cases.
✓ Elevated lipase is associated with worse outcomes in COVID-19 disease.

**TRANSLATIONAL IMPACT**

✓ Assessment for pancreatic injury should be considered in severe cases of COVID-19.
✓ Patients with COVID-19 with elevated lipase could have poor outcome and need to be closely monitored.
